# Primary School Children’s Self-Reports of Attention Deficit Hyperactivity Disorder-Related Symptoms and Their Associations With Subjective and Objective Measures of Attention Deficit Hyperactivity Disorder

**DOI:** 10.3389/fnhum.2022.806047

**Published:** 2022-02-16

**Authors:** Ortal Slobodin, Michael Davidovitch

**Affiliations:** ^1^The Department of Education, Ben-Gurion University, Be’er Sheva, Israel; ^2^Child Development North District, Maccabi Healthcare Services, Tel Aviv-Yafo, Israel; ^3^Kahn-Sagol-Maccabi Research and Innovation Institute, Maccabi Healthcare Services, Tel Aviv-Yafo, Israel

**Keywords:** ADHD (attention deficit and hyperactivity disorder), children, diagnosis, self-report ADHD symptoms, continous performance test

## Abstract

**Background:**

The diagnosis of Attention deficit hyperactivity disorder (ADHD) is primarily dependent on parents’ and teachers’ reports, while children’s own perspectives on their difficulties and strengths are often overlooked.

**Goal:**

To further increase our insight into children’s ability to reliably report about their ADHD-related symptoms, the current study examined the associations between children’s self-reports, parents’ and teachers’ reports, and standardized continuous performance test (CPT) data. We also examined whether the addition of children’s perceptions of ADHD-symptoms to parents’ and teachers’ reports would be reflected by objective and standardized data.

**Methods:**

The study included 190 children with ADHD, aged 7–10 years, who were referred to a pediatric neurologic clinic. A retrospective analysis was conducted using records of a clinical database. Obtained data included children’s self-reports of their attention level and ADHD-related symptoms, parent, and teacher forms of the Conners ADHD rating scales, Child Behavior Checklist (CBCL), Teacher’s Report Form (TRF), and CPT scores.

**Results:**

Children’s self-evaluations of their functioning were globally associated with their teachers’ and parents’ evaluations, but not uniquely. Children’s self-reports of ADHD symptoms were not uniquely linked to a specific CPT impairment index, but to a general likelihood of having an impaired CPT. The CPT performance successfully distinguished between the group of children who defined themselves as inattentive and those who did not.

**Conclusion:**

Primary school children with ADHD are able to identify their limitations and needs difficulties and that their perspectives should inform clinical practice and research. The clinical and ethical imperative of taking children’s perspectives into account during ADHD diagnosis and treatment is highlighted.

## Introduction

Attention deficit hyperactivity disorder (ADHD) is currently one of the most prevalent childhood disorders (Barkley, 2015), with an estimated prevalence of 8.8% in children aged 3–17 years (Centres for Disease Control and Prevention [CDC], 2019). ADHD diagnosis in children and adolescents involves multiple sources of information, with parents and teachers traditionally used as main sources for establishing diagnoses (Wolraich et al., 2011).

Increasingly, research has recognized the importance of children’s and youth’s self-report of ADHD in the diagnosing and treating ADHD (Klimkeit et al., 2006; Ebesutani et al., 2011). However, because research on the utility of self-report measures of ADHD is mainly focused on adolescents, little is known about how younger children perceive their abilities and disabilities and whether these perceptions adhere to the consensus among researchers, professionals, parents, and teachers about the behaviors that characterize ADHD.

The current study aimed to examine whether and how children’s self-report of ADHD-related symptoms are associated with parents’ and teachers’ reports as well as with objective and standardized measures of ADHD (Continuous performance test; CPT). The study also examined whether the addition of children’s perceptions of ADHD symptoms to parents’ and teachers’ reports would be reflected by objective and standardized data.

## Concordance of Self-And Informant-Ratings of Attention Deficit Hyperactivity Disorder Symptoms in Children

Historically, ADHD practice and research assumed that “children’s own descriptions of their problems are not a necessary or particularly useful part of the assessment procedure” (Bierman, 1983, p. 217) and that adults provide more reliable information about a child’s behavior than a child’s self-report (Hoza et al., 2002, 2004).

Even today, research focusing on self-report measures of ADHD is greatly limited to adolescents and young adults. Most existing measurements assessing children’s self-report of ADHD, such as the youth self-report (YSR for ages 11–18 years; Ebesutani et al., 2011), are assigned for adolescents and pre-adolescents, though others, such as the Conners 3rd version (Conners, 2008) and Self-Evaluation Scale for children (Klimkeit et al., 2006) address younger children.

Consistent with studies in adolescents and adults (Pierrehumbert et al., 2006; Kooij et al., 2008), studies of children with ADHD showed that despite chronic functional problems in academic, social, and behavioral domains, many children with ADHD tend to underestimate and underreport their difficulties while overestimating their competencies (e.g., Houghton et al., 2015; Capodieci et al., 2019). This phenomenon has been termed the positive illusory bias (PIB) and is operationally defined as a disparity between self-report of competence and actual competence such that self-reported competence is substantially higher than actual competence (Hoza et al., 2002).

The literature offers several theoretical and methodological explanations for PIB. First, PIB may reflect ADHD-related deficits in metacognitive functions, such as self-reflection, self-awareness, and self-regulation (Zucker et al., 2002; Butzbach et al., 2021). Related to the deficits in meta-cognition is the ability to detect and correct errors. Several studies have highlighted that ADHD is associated with abnormalities in behavioral and neural responsiveness to performance errors. For instance, O’Connell et al. (2009) have demonstrated that children with ADHD usually make significantly more errors than the control group but are less likely to consciously detect these errors. Second, children with ADHD attempt to hide their difficulties by inflating reports of self-competence to prevent feelings of failure or inadequacy and to protect their self-image (Evangelista et al., 2008). The self-protection hypothesis is consistent with the findings of Hoza et al. (2002, 2004), who found that children with ADHD overestimated their competence the most in the domain of greatest deficit.

Third, inaccurate reporting of ADHD-related symptoms in children may be explained by methodological constraints of self-report measurements. Previous studies examining positive self-perceptions in children with ADHD often use a discrepancy score as a measure of positive bias, which is calculated by subtracting the parent’s or teacher’s rating of competency, or alternatively, standardized achievement measures (Owens and Hoza, 2003) from the child’s self-rating. Though discrepancies are useful in examining the extent of overestimation, this method does not consider the unique roles of children’s, parents’, and teachers’ ratings of competency (McQuade et al., 2011). Moreover, previous studies addressing children’s self-perceptions did not measure ADHD symptoms directly but assessed a more global self-evaluation, including perceived scholastic competence, social acceptance, athletic competence, physical appearance, behavioral conduct, and self-worth (McQuade et al., 2011).

Finally, PIB may be attributed to floor or ceiling effects (Owens et al., 2007). Due to the true impairments on the part of children with ADHD, the criterion scores (e.g., actual achievement scores) will almost certainly be much lower for children with ADHD than for control children (Owens et al., 2007). As a result, children with ADHD are more likely to overestimate their competence compared to children without ADHD.

## Concordance of Self-Report Ratings of Attention Deficit Hyperactivity Disorder Symptoms and Continuous Performance Test Performance

While studies converge in suggesting that children’s self-report of ADHD shows lower validity than parent-report (Du-Rietz et al., 2016), the association between children’s self-report of ADHD and laboratory measures of ADHD (e.g., CPT), is largely unstudied.

CPT provides a more objective alternative to reliance on subjective and retrospective recall, thereby reducing the biases that are traditionally associated with self-report measures (Ogundele et al., 2011). Research comparing adults’ self-report measures of ADHD and CPT data revealed mixed results (Baggio et al., 2020). Willard et al. (2016), who examined adolescent survivors of cancer (age 12–17 years), found that self-ratings of ADHD symptoms on the Conners-3, but not parent’s or teacher’s ratings, were associated with CPT performance. In a similar vein, a recent study by Darling (2020) showed that the CPT significantly predicted self-reported symptoms of inattention, hyperactivity-impulsivity, and combined ADHD in adults, over and above measures representing general attention. Other studies, however, showed only limited associations between self-report measures of ADHD and CPT performance. In one study of 201 adults diagnosed with ADHD (based on clinical interview and self-report measures), only 51.7% of the participants were classified as having the disorder based on their CPT performance (Baggio et al., 2020). Similarly, Du-Rietz et al. (2016), who compared self- and parent ratings of ADHD and CPT performance in adolescents and young adults, found that self-reported impairment correlated significantly with fewer objective measures than parent-reported impairment. These results suggested that adolescents and young adults evaluate their level of impairment based on other factors than their parents. Moreover, this study found that although self-reported impairment significantly correlated with several objective measures of ADHD, CPT was far better at distinguishing between ADHD persistent and remittent adolescents and young adults when these were based on parent-report, compared to self-report. Taken together, these findings emphasize the importance of systematically examining children’s self-report ratings of their ADHD-related symptoms and how they correlate with other objective and subjective measures of ADHD.

## The Value of Self-Report Measures of Attention Deficit Hyperactivity Disorder in Children

Despite the documented biases in children’s self-report of their ADHD symptoms, growing evidence has challenged the assumption that children cannot understand or provide information about their own behaviors and own internal states (Owens et al., 2000; Klimkeit et al., 2006; Thorell and Dahlström, 2009). For example, a study by Lufi and Parish-Plass (1995) found that 7–13-year-old children with ADHD rated themselves differently than typically developed children on self-report questionnaires assessing locus of control, anxiety, and persistence. The children with ADHD rated themselves as having a higher external locus of control, less persistence, and more problems with concentration and social relationships. In the same vein, a study focusing on sleep problems showed that children with ADHD reported more sleep disturbances than controls and that their reports were more correlated with their parents’ than those of controls (Owens et al., 2000). Similarly, Klimkeit et al. (2006) found that compared to children without ADHD, children with ADHD report more disorganized, disruptive, and impulsive behaviors; poorer self-perception; and poorer social and communication skills. However, they did not report any less interest in school activities nor more anxiety than the children without ADHD.

Taking children’s self-perceptions into account when diagnosing and treating ADHD may be especially important for girls, who are often under-identified by parents and teachers due to sex-specific biases and expectations (Meyer et al., 2017; Mowlem et al., 2019). Prior studies indicated that the threshold for referral and diagnosis of ADHD in girls might be higher than for boys, mostly because they have fewer hyperactive/impulsive symptoms and more inattentive symptoms compared to boys with ADHD (Willcutt, 2012, for meta-analysis). However, there is evidence to suggest that women with ADHD report more severe ADHD symptoms than men (Vildalen et al., 2019). These results posit that problems in females may have been left undetected and that ADHD symptoms in adulthood may be experienced as more problematic for females than for males due to impaired social behavior (Mikami and Lorenzi, 2011).

The above literature suggests that children’s perceptions of their ADHD-related difficulties and strengths should be addressed by clinicians and researchers, and interventions may be planned accordingly. Taking children’s perceptions of their ADHD symptoms is crucial not only due to their contribution to ADHD diagnostic process but also because inaccurate self-perception may have a detrimental effect on a child’s well-being and future development (Hoza et al., 2001; McQuade et al., 2011). Previous research suggested that while overestimation of competencies may buffer children with ADHD from the effects of failure experiences and protect their self-esteem (Diener and Milich, 1997; Owens et al., 2007), it may also prevent adaptive coping with ADHD-related difficulties, especially if not correlated with improved persistence, motivation, or performance at a task. Therefore, early assessment and intervention promoting accurate self-perception and meta-cognitive skills are crucial (Rizzo, 2011).

## The Current Study

Previous research on children’s self-reports of their ADHD-related symptoms has largely compared adolescents’ perceptions to parent or teacher perceptions, using a discrepancy score as a measure of positive bias (Du-Rietz et al., 2016). This approach assumes that parents’ and teachers’ reports are less biased than self-report ones, overlooking the vulnerability of these methods to informant biases (Rousseau et al., 2008). By contrast, our focus was the comparison between perceptions of competencies and difficulties and standardized CPT data. Thus, we sought to answer whether perceptions of ADHD children are “biased” away not only from other informants but also from standardized measures. The current study used the CPT, which is currently the most popular laboratory-based measure of ADHD in children and adults (Vogt and Williams, 2011; Fuermaier et al., 2019).

The main objectives of the present study are therefore to examine (a) whether gender differences exist between boys and girls in self-reports of ADHD symptoms (b) whether children’s self-reports of ADHD symptoms are in agreement with parents’ and teachers’ reports (b) the pattern of correlations between children’s self-reported ADHD symptoms and CPT performance (c) whether the addition of children’s perceptions of ADHD symptoms to parents’ and teachers’ reports would be reflected by their CPT performance.

## Materials and Methods

### Participants and Procedure

The current study included 190 children diagnosed with ADHD (59.5% male), referred to a private pediatric neurologic clinic. Families are entitled to partial reimbursement. Therefore, the clinic was accessible for patients from heterogenic socio-economic backgrounds. Children were referred to the clinic for ADHD evaluation between January 2018 and December 2020. Participating children and their families were all of Jewish background, living in rural and urban areas in the North of Israel.

Children’s age ranged between 7 (0 month) and 10 (0 month) years (Mean = 8.48, *SD* = 0.90). The diagnostic procedure of ADHD was conducted by a certified pediatric neurologist (the second author) and included an interview with the child and the parents, medical/neurological examination, CPT administration, and ADHD diagnostic questionnaires. Clinical interviews and examination screened for the existence of other conditions and disorders and excluded children who were found unsuitable for the study (see exclusion criteria).

Diagnosis of ADHD was considered positive if, based on both parents’ and teachers’ reports (Conners, 2008), the child scored above the standard clinical cut-offs for ADHD symptoms. Since this is a clinical setting, a more conservative cut-off (+ 2 Standard deviations and above) for ADHD diagnosis was used (Barkley, 2015).

Exclusion criteria were an intellectual disability, severe neurological or developmental disabilities (e.g., cerebral palsy, autism spectrum disorder), and psychosis.

The protocol for the research project conforms to the provisions of the Declaration of Helsinki, approved by the Institutional Review of the Board of Maccabi healthcare services (0181-20-MHS).

### Measurements

Background variables included the child’s age, gender, and grade level.

ADHD-related symptoms were assessed by the parent and teacher forms of the Conners ADHD Index Rating scales, 3rd edition, short-form (Conners 3 AI; Conners, 2008), Hebrew version (Psychtech Ltd, 2012). The Conners 3 is a multi-informant assessment of children between 6 and 18 years of age that takes into account home, social, and school settings and is considered to be a reliable instrument for detecting ADHD problems in children aged 6–18 years. The parent version has content scales for measuring inattention (five items), hyperactivity/impulsivity (six items), learning problems (five items), executive functioning (five items), aggression (five items), and peer relations (five items). The teacher version has five content scales, measuring inattention (five items), hyperactivity/impulsivity (six items), learning problems/executive functioning, aggression (five items), and peer relations (five items). Items are rated on a Likert-type scale of 0 (not true at all), 1 (just a little true), 2 (pretty much true), or 3 (very much true). The standard rating period is 1 month for both versions.

The psychometric properties of the Conners 3 scales have been previously tested and confirmed in various cultural contexts (Morales-Hidalgo et al., 2017; Izzo et al., 2018). Results of reliability analyses revealed that the Conners 3 forms have high levels of internal consistency (mean Cronbach’s alpha = 0.90) and excellent test-retest reliability (mean *r* = 0.83). The Conners 3 manual also reports high convergent/divergent validity through correlations with other related measures of childhood psychopathology and high utility in discriminating between relevant groups. In terms of the classification accuracy of the scores (as determined by a series of discriminant function analyses), the mean overall correct classification rate was 75.6% (Conners et al., 2011).

Co-existing psychiatric symptoms were measured by the Child Behavior Checklist (CBCL), and the Teacher’s Report Form (TRF; Achenbach and Rescorla, 2001), Hebrew version (Psychtech Ltd, 2005). These forms include eight DSM-oriented scales consistent with DSM diagnostic categories: Anxious/Depressed, Withdrawn/Depressed, Somatic Complaints, Social Problems, Thought Problems, Attention Problems, Rule-Breaking Behavior, and Aggressive Behavior. The 120 items on the CBCL are rated on a Likert-type scale of Not True (0), Somewhat or Sometimes True (1), or Very True or Often True (2). Validity and reliability of the scales have been documented and extensive normative data are available for children ranging from 6 to 18 (Achenbach and Rescorla, 2001). Previous studies have demonstrated that both the CBCL and the TRF showed high utility in distinguishing between children with and without ADHD diagnosis (Eiraldi et al., 2000; Edwards and Sigel, 2015). For example, for a mixed group of clinic-referred and general community children, Lampert et al. (2004) reported an area under the curve (AUC) of 0.79, and sensitivity and specificity at the optimum cutoff (raw score of 9) of 75 and 70%, respectively. A recent study by Gomez et al. (2021), using receiver operating characteristic curve analysis (ROC), showed that the abilities of the CBCL and the TRF attention problems scales for identifying ADHD in 6–11 years old children, were very good (AUC = 0.86 and 0.75, respectively).

Perceived level of attention—children were asked to report their level of attention during class, whereas “1” indicates the lowest attention level and “10” is the highest. To ensure consistency with teachers’ and parents’ scales, where higher scores indicate greater pathology, we reversed children’s scores during data analysis, so that “1” indicated the highest attention score and “10” the lowest. Since children’s self-reports of comorbid symptoms were rated on three levels of severity, we decided to categorize the perceived attention scale into three levels as well to present a more coherent picture of our findings. We have, therefore, coded the reported scores into three levels of attention: scores ranging from 1 to 3 were coded as “high attention level,” scores between 4 and 6 were coded as “medium attention level” and scores equal or higher than 7 as “poor attention level.”

Children’s self-report of comorbid symptoms—Based on the Diagnostic and Statistical Manual of Mental Disorders (DSM; American Psychiatric Association, 2013) as well as on clinical experience, this scale included five comorbid symptoms of ADHD, including social problems, learning difficulties, school aversion, anxiety, and depression. The five comorbid symptoms of ADHD largely parallel the symptoms that appear in the CBCL and the TRF, addressing social problems, learning difficulties, anxiety, and depression. However, to overcome social desirability we asked children to report more generally about school aversion and not directly about aggression and rule-breaking.

Given previous research indicating that school children have a preference for questions rather than statements (Royeen, 1985), difficulties in each domain were assessed by presenting a question to the child (e.g., “how good are you with friends?” or “How satisfied are you with your social status?”). On each item, children’s answers were scored into one of three levels, with a higher score reflecting increased distress.

CPT performance—the study employed the MOXO-CPT^1^ version (Berger and Goldzweig, 2010), a standardized continuous test designed to assess ADHD-related symptoms. Like other CPTs, the MOXO-CPT measures sustained attention, omission and commission errors, and response time, but is also able to differentiate between different types of disinhibited responses and between problems in response time and inattention. Furthermore, the test includes external auditory and visual interfering stimuli serving as measurable distractors. The test’s validity and utility in distinguishing children and adolescents with ADHD from their typically developing peers were demonstrated in previous studies (Berger and Cassuto, 2014; Berger et al., 2017; Shahaf et al., 2018; Slobodin et al., 2020). A detailed description of the test’ stimuli, distractors, levels can be found in Supplementary Material 1.

The MOXO-CPT measured four performance indices:

Attention: the number of correct responses (pressing the key in response to a target stimulus), conducted either during the stimulus presentation or during the void period that followed. This method allows the test to evaluate whether the participant was attentive to the target independently of his/her response time. The number of omission errors was also calculated (i.e., the number of times that the patient did not respond to a target stimulus).

Timing: the number of correct responses (pressing the key in response to a target stimulus) conducted while the target stimulus was still presented on the screen. This index excluded responses that were performed during the void period (after the stimulus has disappeared). This method allowed the test to differentiate between the overall rate of correct responses (measured by the Attention index) and the rate of correct responses that were conducted on time (measured by the Timing index). These two aspects of timing correspond to two different deficits typical to ADHD: difficulty to provide an accurate response and difficulty to respond on time (National Institute of Mental Health, 2012).

Impulsivity: the number of commission errors conducted while a non-target stimulus was present on the screen. Other types of non-inhibited responses (e.g., pressing the keyboard more than once) were not considered as impulsive responses (as will describe in the next paragraph).

Hyperactivity: the total number of commission responses that were not coded as impulsive responses (e.g., multiple responses, random key pressing). Differentiating between commission errors that were conducted due to impulsive behavior and commission errors that were conducted due to motor hyper-responsivity allowed the identification of multiple sources of response disinhibition.

The children’s version of the MOXO-CPT showed high specificity and sensitivity rates (≥85%) in children aged 7–12 years (Berger et al., 2017). AUC values for the attention index ranged between 0.75 and 0.91, for the timing index between 0.80 and 0.90, and the hyperactivity index between 0.73 and 0.82, depending on the child’s age. The impulsivity parameter, however, showed AUC between 0.58 and 0.65, indicating low diagnostic performance when considered alone. The MOXO-CPT total score, which integrates all four CPT indices, consistently showed excellent ability to discriminate children with and without ADHD for all observed ages, with AUC values above 0.91. In the current study, we found that the Attention index was negatively correlated with the Impulsivity (*r* = −0.274, *p* < 0.01) and the Hyperactivity (*r* = −0.204, *p* < 0.01) indices. The Impulsivity index was highly and positively correlated with the Hyperactivity index (*r* = 0.700, *p* < 0.01). The Timing index was not significantly correlated with the Attention (*r* = −0.19, *p* = 0.796), Impulsivity (*r* = −0.46, *p* = 0.535), or the Hyperactivity (*r* = −0.48, *p* = 0.521) indices.

### Data Analysis

Gender differences in children’s self-report of ADHD- related symptoms were examined with chi-square tests. To examine whether children’s self-reports of ADHD symptoms are in agreement with parents’ and teachers’ reports, we conducted a spearman correlation matrix between study variables. Further, to examine the pattern of correlations between children’s self-reported ADHD symptoms and CPT performance, we conducted a spearman correlation matrix between children’s self-report of attention and ADHD-comorbid symptoms and (a) each of the four MOXO-CPT indices (Attention, Timing, Hyperactivity, and Impulsivity), (b) the number of impaired CPT indices (ranging from 1 to 4), and (c) the presence of at least one impaired CPT index (yes/no). The child’s attention profile presents his or her performance level in relation to the norm group (reflecting the corresponding age and gender) for each index. A standard score is calculated for each index and categorized into one of four possible performance levels. The MOXO-CPT considers an index to be impaired if the child obtained a Z score ≥ 1.6.

Finally, to examine whether the addition of children’s self-reports of ADHD symptoms to informant reports is reflected by CPT data, we divided children into three distinct groups based on teacher, parent, and child reports; (1) The parent’s or the teacher’s scores on inattention and/or hyperactivity scales of the Conners were elevated (single informant). We considered children to be at risk of ADHD if they obtained a *T*-score ≥ 65. This cut-off score was chosen to include all the subjects with elevated and very elevated scores (Conners, 2008).

(2) Both parent and teacher reported elevated levels of ADHD symptoms (two informants). (3) Parent and/or teacher reported elevated levels of ADHD symptoms and the child reported poor attention (i.e., scored 7 or higher). We assumed that children whose self-reports of inattention were in the upper third of the scale had the highest subjective severity of ADHD symptoms.

Group differences in MOXO-CPT performance were examined using ANOVA, followed by *post hoc* analyses with Tukey correction for multiple comparisons. Analyses were conducted with SPSS software for Windows Version 25 (SPSS, Inc., Chicago, IL, United States).

## Results

Table 1 presents children’s self-report of ADHD symptoms for boys and girls. As seen, moderate magnitudes of inattention, anxiety, and learning difficulties were reported by both boys and girls. Disliking school was frequently reported. Social problems were less frequently reported. No gender differences were observed in the examined variables.

**TABLE 1:** Children’s self-report of ADHD-related symptoms, by gender.

Problem severity	Low		Moderate		High		Difference
	Boys	Girls	Boys	Girls	Boys	Girls	
	N (%)	N (%)	N (%)	N (%)	N (%)	N (%)	
Attention problems	21 (21)	11 (15.5)	58 (58)	47 (66.2)	21 (21)	13 (18.3)	(^2^(2) = 1.02, *p* = 0.600
Social problems	70 (64.2)	54 (72)	25 (22.9)	13 (17.3)	14 (12.8)	8 (10.7)	(^2^(2) = 1.25, *p* = 0.535
Leaning difficulties	17 (15)	12 (15.8)	79 (61.1)	53 (69.7)	27 (23.9)	11 (14.5)	(^2^(2) = 2.552, *p* = 0.279
Dislike school	30 (26.8)	23 (29.9)	31 (27.7)	26 (33.8)	51 (45.5)	28 (36.4)	(^2^(2) = 1.634, *p* = 0.492
Anxiety	44 (44.4)	21 (29.6)	34 (34.3)	35 (49.3)	21 (21.2)	15 (21.1)	(^2^(2) = 4.668, *p* = 0.097
Depression	70 (62.5)	45 (58.4)	38 (33.9)	31 (40.3)	4 (3.6)	1 (1.3)	(^2^(2) = 1.515, *p* = 0.469

Table 2 presents the Spearman correlation matrix between children’s self-reported ADHD symptoms and teacher and parents reports. Results indicated that children’s self-reports of inattention were positively correlated with parents’ reports of inattention (*r* = 0.179, *p* < 0.05) and hyperactivity (*r* = 0.246, *p* < 0.01). Also, children’s self-reports of inattention correlated with teachers’ reports of social problems (*r* = 0.174, *p* < 0.05). Children’s self-reports of social problems were positively associated with parents’ report of social problems (*r* = 0.206, *p* < 0.01) and anxiety (*r* = 0.164, p < 0.05), and with teachers’ reports of social problems (*r* = 0.283, *p* < 0.01) and depression (*r* = 0.270, *p* < 0.01). Children’s self-reports of learning difficulties were positively and significantly associated with teachers’ reports of inattention (*r* = 0.167, *p* < 0.05) and learning difficulties (*r* = 0.213, *p* < 0.01). Children’s self-reports of anxiety were correlated only with parental reports of anxiety (*r* = 0.178, *p* < 0.05). Finally, children’s self-reports of depression were associated with parental reports on child’s social problems (*r* = 0.172, *p* < 0.05), and anxiety (*r* = 0.234, *p* < 0.01) and with teachers’ reports on social problems (*r* = 0.209, *p* < 0.01), anxiety (*r* = 0.153, *p* < 0.05), and depression (*r* = 0.185, *p* < 0.05).

**TABLE 2:** Spearman correlations matrix between self-, parent-, and teacher- reports of ADHD-related symptoms.

		Children’s self-reports					
		Attention problems	Social problems	Leaning difficulties	Dislike school	Anxiety	Depression
**Parents’ reports**	Attention problems (Conners)	0.179*	−0.094	−0.124	−0.100	−0.102	0.099
	Hyperactivity (Conners)	0.246**	−0.020	−0.009	0.034	−0.066	0.049
	Learning difficulties (Conners)	0.076	−0.009	0.061	0.001	0.118	0.022
	Social problems (CBCL)	0.068	0.206**	−0.081	0.020	0.046	0.172*
	Anxiety (CBCL)	0.054	0.164*	0.009	0.108	0.178*	0.234**
	Depression (CBCL)	−0.046	0.133	0.009	0.056	−0.057	0.137
Teachers’ reports	Attention problems (Conners)	0.138	−0.084	0.167*	0.039	0.063	−0.061
	Hyperactivity (Conners)	0.124	0.013	0.104	0.066	−0.006	0.047
	Learning difficulties (Conners)	0.021	−0.015	0.213**	0.043	0.105	−0.011
	Social problems (TRF)	0.174*	0.283**	0.029	0.123	0.114	0.209**
	Anxiety (TRF)	0.031	0.113	0.069	0.040	0.147	0.153*
	Depression (TRF)	0.026	0.270**	0.102	0.107	0.066	0.185*

*p < 0.05; **p < 0.01.

Table 3 presents the Spearman correlation matrix between children’s self-reported ADHD symptoms and MOXO-CPT performance. Results showed that children’s self-reports of inattention were associated with an increased likelihood of obtaining at least one impaired CPT index (*r* = 0.211, *p* < 0.01). Self-reports of learning difficulties were negatively associated with children’s ability to respond on accurate timing during the CPT (*r* = −0.162, *p* < 0.05), and positively with the number of impulsive responses (*r* = 0.212, *p* < 0.01). Self-reports of disliking school were negatively associated with children’s ability to respond on accurate timing during the CPT (*r* = −0.185, *p* < 0.05), but positively with the likelihood of obtaining at least one impaired CPT index (*r* = 0.202, *p* < 0.01).

**TABLE 3:** Spearman correlations matrix between self-report of ADHD-related symptoms and impaired MOXO-CPT indices.

	Children’s self-reports					
	Attention problems	Social problems	Leaning difficulties	Dislike school	Anxiety	Depression
MOXO-A	0.014	0.030	−0.030	−0.142	0.058	0.036
MOXO-T	0.137	−0.051	−0.162*	−0.185*	0.055	−0.015
MOXO-I	0.098	0.010	0.212**	0.030	0.008	0.085
MOXO-H	0.074	0.025	−0.038	−0.115	0.063	0.079
Number of impaired indices	0.124	0.020	−0.027	−0.142	0.066	0.096
At least one impaired CPT index	0.211**	−0.013	−0.120	0.202**	0.081	0.002

*p < 0.05; **p < 0.01.

*MOXO-A, Attention index; MOXO-T, Timing index; MOXO-I, Impulsivity index; MOXO-H, Hyperactivity index. In the Attention and Timing indices, higher scores mean better performance. In the Hyperactivity and Impulsivity indices, higher scores mean worse performance (increased hyperactive and impulsive responses).*

In the current study, children’s self-report on co-morbid symptoms were rated on a three-point Likert scale and not on a scale ranging from 1 to 10 as the perceived attention item. To present a more coherent picture of our findings, we decided to categorize the perceived attention item into three levels as well. Nevertheless, an examination of the relationship between the continuous variable of the child perceived attention, parent/teacher, and CPT data showed only a slight difference from the categorized variable; the continuous variable of child’s perceived attention, but not the categorized one, correlated significantly with teachers’ ratings of hyperactivity (*r* = 0.155, *p* < 0.05). In all other analyses, the results for the continuous variable were very similar to that of the categorized one (Pearson correlations values are presented in Supplementary Material 2).

Group differences in MOXO-CPT indices are presented in Table 4. ANOVA analysis showed that the only significant difference between the three groups (parent or teacher reported elevated levels of ADHD symptoms, both parent or teacher reported elevated levels of ADHD symptoms, and child’s report of inattention with either parent, teacher, or both) was in their likelihood of having at least one impaired CPT index. That is the risk of having impaired CPT performance was significantly higher in children who reported having problems of inattention compared to children who did not.

**TABLE 4:** Group differences in CPT performance.

	Either parent or		Parent and		Child with either parent, teacher,		Difference
	teacher (1) *N* = 54		teacher (2) *N* = 99		or both (3) *N* = 32		
	**Mean**	***SD***	**Mean**	***SD***	**Mean**	***SD***	
MOXO-A	1.515	2.144	1.625	2.267	1.696	2.177	*F*_(182)_ = 0.073, *p* = 0.930
MOXO-T	2.479	1.493	34.054	304.308	3.324	1.659	*F*_(182)_ = 0.436, *p* = 0.648
MOXO-I	1.115	1.933	0.999	1.503	1.633	2.352	*F*_(182)_ = 1.383, *p* = 0.254
MOXO-H	3.098	5.200	2.873	4.198	5.135	6.486	*F*_(182)_ = 2.394, *p* = 0.094
Number of impaired indices	1.722	1.352	1.687	1.283	2.031	1.031	*F*_(182)_ = 0.925, *p* = 0.398
Having at least one impaired CPT index	0.759	0.432	0.778	0.418	0.969	0.177	*F*_(182)_ = 3.40, *p* = 0.035 3 > 2.1

*MOXO-A, Attention index; MOXO-T, Timing index; MOXO-I, Impulsivity index; MOXO-H, Hyperactivity index.*

*In the Attention and Timing indices, higher scores mean better performance. In the Hyperactivity and Impulsivity indices, higher scores mean worse performance (increased hyperactive and impulsive responses).*

## Discussion

Although research has increasingly recognized the importance of patients’ self-perception of ADHD symptoms to the diagnosis and treatment of the disorder (Ustun et al., 2017; Adler et al., 2019), studies focusing on children’s self-reports are severely limited. The current study examined the extent to which children’s self-report of ADHD symptoms are associated with informant reports as well as with standardized measures of ADHD (CPT). Additionally, the study examined whether the addition of children’s self-reports to parents’ and teachers’ reports would be reflected by their CPT performance.

### Agreement Between Self- and Informant Reports

Our results indicated that children’s self-evaluations of their functioning were often associated with their teachers’ and parents’ evaluations. However, these correlations were small to moderate (Cohen, 1988) and not symptom-specific. That is, children’s self-reports of their academic, social, and emotional difficulties were not uniquely associated with the same difficulties according to informant reports. For example, we found that children’s self-report of inattention was associated with parents’ report of inattention and hyperactivity, but with teachers’ reports of social problems. Likewise, we found that children’s self-reports of depression were associated with parental reports on social problems and anxiety and with teachers’ reports on social problems, anxiety, and depression. These results suggest that primary school children with ADHD can recognize and report about their difficulties, and thus provide meaningful and clinically useful information on their academic, social, and emotional functioning (Penza-Clyve and Zeman, 2000; Klimkeit et al., 2006). At the same time, the modest associations between self-report ADHD symptoms and parent/teacher reports emphasize the importance of using multiple sources of information in the diagnostic process of ADHD (Wolraich et al., 2011). Consistent with previous studies, our results may indicate that teacher-, parent-, and self-perceptions each provide unique information that contributes to the comprehensive evaluation of a child (Jardine et al., 2014; Willard et al., 2016).

The relationship between children’s and informant reports is probably bidirectional. Parents and teachers are key figures in a child’s everyday life and therefore have multiple opportunities to learn about a child’s behavior (Bied et al., 2017). Nevertheless, the way parents and teachers evaluate children may affect children’s behavior and their self-perception. Prior studies suggested that everyday interactions with parents and teachers shape children’s perceptions of their strengths and difficulties through their relationship with the child (Eisenberg and Schneider, 2007; Granot, 2016; Leitch et al., 2019). A recent review of the impact of the children-teacher relationship on children with ADHD showed that children were highly sensitive to how they are perceived by their teachers. Moreover, teachers’ rejection of ADHD students was identified as a risk factor for school failure, peer exclusion, and rejection, leading to low self-esteem and loneliness (Ewe, 2019). When parents and teachers have negative perceptions of the child, they tend to express more stress, hostility, and aggression (Lifford et al., 2008), exacerbating the child’s difficulty and leading to a negative vicious cycle.

Our findings raise an important question about whether the agreement between child’s and informant reports varies across contexts (e.g., home, school). Our results demonstrated that children’s self-reports of learning difficulties were linked only to teachers’ reports of inattention and learning difficulties, but not with any parental report measures. According to the literature on informant agreement in childhood ADHD, teachers and parents capture different aspects of a child’s behavior (Geiser, 2009; Slobodin and Davidovitch, 2019). Teachers were often found as a more reliable source of information about ADHD because ADHD-related social and academic difficulties are more prominent in the school environment than at home (Biederman et al., 1995), but also because parents may be more biased than teachers in their ADHD ratings (Hartman et al., 2007). On the other hand, parent ratings continue to be important in adolescence and young adulthood, as they appear to better reflect objective measures of impairment, as well as measures such as the heritability of ADHD (Du-Rietz et al., 2016). Differences in agreement rates between child-parent and child-teacher reports as well as the underlying mechanisms of these agreements require further research.

### Children’s Self-Report and Continuous Performance Test Performance

Correlation analysis between CPT performance indices and children’s self-reports indicated that children’s perceptions of their ADHD are mildly reflected by their CPT performance. Specifically, we found that children’s self-reports of inattention, and disliking school were associated with an increased likelihood of having at least one impaired CPT index. Additionally, self-reports of learning difficulties were associated with increased impulsivity and with decreased ability to respond at an accurate timing. The latter is valid also for self-reports of disliking school. Together, these results suggest that children’s self-reports of ADHD were not uniquely linked to a specific CPT index, but to the general likelihood of having an impaired CPT. Moreover, the fact that impairments in CPT performance were associated with children’s self-reports of their academic-related functioning and not with their emotional or social functioning provides additional support for primary school children’s ability to reliably perceive and report their difficulties.

In line with previous findings in adolescents and adults, our results suggest that children’s self-report of their difficulties and strengths are only mildly and globally associated with their CPT performance. Recent findings of the association between the self-report of ADHD and CPT performance showed that adolescents’ and young adults’ reports of ADHD outcomes were only partly reflected by their CPT (Jarrett et al., 2017; Baggio et al., 2020). To examine whether the addition of children’s perceptions to informant reports would be reflected by objective measures, we compared CPT performance of three groups of children: children whose (1) parent or teacher reported elevated levels of ADHD symptoms (single informant) (2) both parent and teacher reported elevated levels of ADHD symptoms (two informants), and (3) parent and/or teacher reported elevated levels of ADHD symptoms and the child reported poor attention.

Analysis of differences between the groups showed that the three groups did not differ in the type of CPT impairment (impaired CPT index) nor the magnitude of such impairment (the number of impaired indices). However, we found that the risk of having at least one impaired CPT index was significantly higher in the group where both children and at least one informant reported elevated levels of ADHD symptoms. Our findings indicate that while ADHD children defined by one informant showed similar CPT profiles to ADHD children defined by two informants, the CPT performance successfully distinguished between these two groups and the group of children who also defined themselves as inattentive. These results suggest that adding children self -report to the diagnostic process of ADHD may improve the low-medium correspondence between informants’ reports and objective measures of ADHD documented in the literature (Slobodin and Davidovitch, 2019).

This study expanded prior research on the self-perception of children with ADHD in several ways. First, this study is one of the few to examine agreement between primary school children’s self-reports of their ADHD-related difficulties and a standardized objective measure of ADHD. Second, we included both girls and boys, making this one of the very few examinations of self-report in a large clinical sample.

Several limitations of this study should be acknowledged. First, the current study did not allow the calculation of discrepancies between child and informant reports. Therefore, more information is needed about how children’s self-perceptions differ from those of parents and teachers and how these discrepancies are affected by age and gender. Furthermore, although we found that self-reports of ADHD symptoms were only mildly reflected by objective measures, self-reports may be better captured by other measures not included in our study. Second, the fact that we didn’t distinguish between different ADHD subtypes (predominantly Inattentive, predominantly Hyperactive-Impulsive, and Combined) limits our ability to examine whether they are differently related to children’s self-reports. Previous studies suggested that ADHD subtypes may be subject to different perception biases (Owens and Hoza, 2003). For example, children with the inattentive subtype of ADHD tend to exhibit negatively biased self-perceptions (Owens and Hoza, 2003). Thus, further investigation of the validity of children’s self-reports and their added value to the diagnosis of ADHD is of high importance.

Third, the current study assessed children’s perceived attention level using a single item that asked them to report about their general ability to stay attentive and concentrated during class. Probably, children’s answers reflected attention problems, hyperactivity/impulsivity symptoms, and their distractibility level. Using a single item limits the psychometric accuracy of this measure and hinders our ability to examine specific associations between different children’s self-report of ADHD symptoms, parent/teacher reports, and CPT data. We, therefore, encourage future studies to identify how different self-report ADHD symptoms are reflected by other objective and subjective measures of ADHD.

Finally, the generalizability of our results is limited, given the ethnic and geographic homogeneity of the sample, its limited size, and the fact that all children were recruited from a single neuro-pediatric clinic. Our ability to generalize our findings is also limited due to the restricted age range of participating children. The effects of the child’s age on the validity of self-report ADHD are currently understudied. However, there is evidence that children’s metacognitive abilities that allow them to identify and report about their performance and experiences can already be evident in toddlers and tend to increase with age (Riley, 2004; Harter, 2012). For example, Varni et al. (2007) showed that even preschool children can reliably and validly self-report about their well-being when given the opportunity to do so with an age-appropriate instrument. A child’s age may also influence teachers’ and parents’ ratings, as the time spent with individual teachers usually reduces with maturation. Depending on the subject matter and the interest level of the student, ADHD symptoms may not be as observable during limited periods (Willard et al., 2016). In contrast, parent ratings are likely influenced by an extended period of observation over many years. Future studies should examine the validity of children’s self-report of ADHD symptoms in various age groups.

At a practical level, we found that primary school children are clinically informative as to what to focus our interventions on for each child (i.e., social skills training, self-esteem building) as well as providing some insight into the presence of ADHD symptomatology.

Further investigations into self-reported impairment and its correlates would be beneficial to understand on what basis young individuals estimate their levels of impairment. Regardless of their predictive validity, taking children’s perspectives into account while diagnosing and treating ADHD may have significant clinical outcomes. Being curious about how children perceive and cope with their difficulties promotes productive, respectful, and empathic interactions between children, parents, and professionals, and may eventually contribute to their collaboration and treatment adherence (Brinkman et al., 2018). Moreover, children’s self-reports are essential for monitoring treatment outcomes. Although many initiatives have created the opportunity for children to be included in clinical trials, children’s perspectives about their well-being were often overlooked when evaluating the efficacy of treatments (Varni et al., 2007). The U.S. Food and Drug Administration (FDA) stated that “some treatment effects are known only to the patient” and recommended that instrument development and validation testing for children and adolescents be conducted within fairly narrow age groupings to determine the lower age limit at which children can provide reliable and valid responses (FDA, 2006).

Finally, questioning children about their ADHD-related difficulties may enhance their self-awareness and improve their ability to reflect upon their difficulties and strengths.

## Data Availability Statement

The raw data supporting the conclusions of this article will be made available by the authors, without undue reservation.

## Ethics Statement

The studies involving human participants were reviewed and approved by the Maccabi Health Services Ethics Committee. Written informed consent for participation was not required for this study in accordance with the national legislation and the institutional requirements.

## Author Contributions

MD collected clinical information. OS wrote the original draft. Both authors have equally contributed to the conceptualization, methodology, analysis, and interpretation.

## Conflict of Interest

The authors declare that the research was conducted in the absence of any commercial or financial relationships that could be construed as a potential conflict of interest.

## Publisher’s Note

All claims expressed in this article are solely those of the authors and do not necessarily represent those of their affiliated organizations, or those of the publisher, the editors and the reviewers. Any product that may be evaluated in this article, or claim that may be made by its manufacturer, is not guaranteed or endorsed by the publisher.
