# Supplementary material for: Primary School Children’s Self-Reports of Attention Deficit Hyperactivity Disorder-Related Symptoms and Their Associations With Subjective and Objective Measures of Attention Deficit Hyperactivity Disorder
Source: Front Hum Neurosci. 2022 Feb 16;16:806047. doi: 10.3389/fnhum.2022.806047 (PMC8888855; doi:10.3389/fnhum.2022.806047)
Supplement: Supplementary file 1 [file Data_Sheet_1.docx]

**Supplementary 1: The MOXO Continuous Performance Test (CPT)** The study employed the MOXO-CPT^^[[1]](#footnote-1)^^ version (Berger and Goldzweig, 2010), a standardized computerized test designed to diagnose ADHD-related symptoms. Like other CPTs, the MOXO-CPT measures sustained attention, omission and commission errors, and response time. However, as detailed below, it differs from other CPTs in its ability to differentiate between different types of disinhibited responses and between problems in response time and inattention. Importantly, the test incorporates external interfering stimuli (auditory and visual) serving as measurable distractors, a feature that is unique to the MOXO-CPT. The test’s validity and utility in distinguishing children and adolescents with ADHD from their typically developing peers were demonstrated in previous studies (Berger et al., 2017; Berger and Cassuto, 2014; Shahaf et al., 2018).

*Test levels.* The test included eight levels (stages), each consisting of 53 trials (33 target and 20 non-target stimuli) and lasted 114.15s. The total duration of the test was 15.2 min. On each trial, a stimulus (target or non-target) was presented in the middle of the screen for 0.5, 1, or 3 seconds and was followed by a “void” of the same length. Each stimulus remained on the screen for the full presentation time, regardless of whether a response was provided or not. This practice allows the measuring of response time as well as its accuracy. The child was instructed to respond to the target stimulus as quickly as possible by pressing the space bar once and only once. In addition, the child was instructed not to respond to any other stimuli but the target, and not to press any other key but the space bar.

*Test stimuli*. Target and non-target stimuli were cartoon pictures. Given that ADHD often co-occurs with specific learning disabilities that may be confounded with CPT performance, all stimuli were free of letters or numbers (Seidman et al., 2001). Target stimulus was always a cartoon image of a child’s face. Non-target stimuli included five different images of animals.

*Distracting stimuli*. To improve the test’s ecological validity and to simulate the everyday environment, the MOXO-CPT incorporated visual and auditory distracting stimuli, that were not part of the non-target stimuli. Distractors’ onset was not synchronized with the onset of the target or the non-target stimuli.

Distractors were short animated video clips with typical elements of the child’s everyday life. Overall, six different distractors were presented, each of them could appear as pure visual (e.g., birds moving their wings), pure auditory (e.g., birds singing), or as a combination of visual and auditory stimuli (birds singing and simultaneously moving their wings). Distractor presentation time varied between 3.5 and14.8 s, with a fixed interval of 0.5 s between two distractors. Distractors were presented continuously between level 2 and level 7 of the test. Visual distractors include six different stimuli: a gong (presented for 6.8s), a bowling ball (3.5s), birds (9.25s), a warrior (Jedi) with saber (14.8s), a saber (6.8s), and a flying airplane (8.6s).

Auditory distractors included the six corresponding sounds of the visual distractors.

*Test levels*. The test included 8 levels, each included different distractors set: levels 1 and 8 did not include any distractors. Levels 2 and 3 included pure visual stimuli, levels 4 and 5 included pure auditory stimuli, and Levels 6 and 7 included a combination of visual and auditory stimuli. During levels 2, 4, and 6, only one distractor was presented at a time. During levels 3,5 and 7, two distractors were presented simultaneously.

*Performance indices*—The MOXO-CPT measured four performance indices:

Attention- The number of correct responses (pressing the key in response to a target stimulus), which were conducted either during the stimulus presentation or during the void period that followed. This method allows the test to evaluate whether the participant responded correctly to the target (was attentive to the target) independently of his/her response time. The number of omission errors was also calculated (i.e., the number of times that the patient did not respond to a target stimulus). The score in the Attention index was calculated as the average of correct responses throughout the eight test levels.

Timing- The number of correct responses (pressing the key in response to a target stimulus) that were given while the target stimulus was still presented on the screen. This index excluded responses that were performed during the void period (after the stimulus has disappeared). This method allowed the test to differentiate between the overall rate of correct responses (measured by the Attention index) and the rate of correct responses that were given only on good timing (measured by the Timing index). These two aspects of response time correspond to two different deficits typical to ADHD: a difficulty to provide an accurate response and difficulty to respond on time (National Institute of Mental Health, 2012).

The score in this index was calculated as the average of correct responses while the target stimulus was still presented on the screen throughout the eight test levels.

Impulsivity - The number of commission errors performed only when a non-target stimulus was present on the screen. Other types of non-inhibited responses (e.g., pressing the keyboard more than once) were not considered as impulsive responses (as will describe in the next paragraph). Score in this index was calculated as the average of impulsive responses throughout the eight test levels.

Hyperactivity- The total number of commission responses that were not coded as impulsive responses (e.g., multiple responses, random key pressing). Differentiating between commission errors that were conducted due to impulsive behaviour and commission errors that were conducted due to motor hyper-responsivity allowed the identification of multiple sources of response disinhibition. The score in this index was calculated as the average of hyperactive responses in the eight test levels.

**Supplementary 2:** Pearson correlations matrix between children’s self-report of attention (continuous variable), parent- and teacher- reports of ADHD-related symptoms, and CPT data

|  | Children’s self-report of attention problems |  |
| --- | --- | --- |
| Parents’ reports | Attention  problems  (Conners) | .201** |
|  | Hyperactivity  (Conners) | .236** |
|  | Learning difficulties  (Conners) | .108 |
|  | Social problems (CBCL) | .130 |
|  | Anxiety  (CBCL) | .170 |
|  | Depression  (CBCL) | -.048 |
| Teachers’ reports | Attention  problems  (Conners) | .102 |
|  | Hyperactivity  (Conners) | .155* |
|  | Learning difficulties  (Conners) | -.002 |
|  | Social problems (TRF) | .200** |
|  | Anxiety  (TRF) | .062 |
|  | Depression  (TRF) | .060 |
| MOXO-CPT | MOXO-A | .048 |
|  | MOXO-T | -.066 |
|  | MOXO-I | .088 |
|  | MOXO-H | .057 |
|  | Number of impaired indices | .110 |
|  | Having at least one impaired CPT index | .158* |

**p*<.05; ***p*<.01; ****p*<.001

1. The term 'MOXO' derives from the world of Japanese martial arts and means a 'moment of lucidity'. It refers to the moments preceding the fight, when the warrior clears his mind from distracting, unwanted thoughts, and feelings. [↑](#footnote-ref-1)
